# A trans-ethnic two-stage polygenetic scoring analysis detects genetic correlation between osteoporosis and schizophrenia

**DOI:** 10.1186/s40169-020-00272-y

**Published:** 2020-02-27

**Authors:** Li Liu, Yan Wen, Yujie Ning, Ping Li, Bolun Cheng, Shiqiang Cheng, Lu Zhang, Mei Ma, Xin Qi, Chujun Liang, Tielin Yang, Xiangding Chen, Lijun Tan, Hui Shen, Qing Tian, Hong-Wen Deng, Xiancang Ma, Feng Zhang, Feng Zhu

**Affiliations:** 1 School of Public Health, Xi’an Jiaotong University Health Science Center, Yanta West Road 76, Xi’an, 710061 People’s Republic of China; 2 Key Laboratory of Biomedical Information Engineering of Ministry of Education, School of Life Science and Technology, Xi’an Jiaotong University, Xi’an, 710049 Shaanxi People’s Republic of China; 3 Laboratory of Molecular and Statistical Genetics, College of Life Sciences, Hunan Normal University, Changsha, People’s Republic of China; 4 Center for Bioinformatics and Genomics, Department of Global Biostatistics and Data Science, School of Public Health and Tropical Medicine, Tulane University, New Orleans, LA USA; 5 Department of Psychiatry, The First Affiliated Hospital of Xi’an Jiaotong University, Xi’an, People’s Republic of China; 6 Center for Translational Medicine, The First Affiliated Hospital of Xi’an Jiaotong University, No. 277 Yanta West Road, Xi’an, 710061 People’s Republic of China

**Keywords:** Osteoporosis, Schizophrenia, Genetic correlation, Genome-wide association analysis

## Abstract

**Backgrounds:**

To explore the genetic correlation between schizophrenia (SCZ) and osteoporosis (OP).

**Design, setting, participants, measurements:**

We conducted a trans-ethnic two-stage genetic correlation analysis of OP and SCZ, totally invoking 2286 Caucasia subjects in discovery stage and 4124 Chinese subjects in replication stage. The bone mineral density (BMD) and bone area values of ulna & radius, hip and spine were measured using Hologic 4500W dual energy X-ray absorptiometry machine. SCZ was diagnosed according to DSM-IV criteria. For the genome-wide association study (GWAS) of Caucasian OP, Chinese OP and Chinese SCZ, SNP genotyping was performed using Affymetrix SNP 6.0 array. For the GWAS of Caucasian SCZ, SNP genotyping was conducted using the Affymetrix 5.0 array, Affymetrix 6.0 array and Illumina 550 K array. Polygenetic risk scoring (PRS) analysis was conducted by PRSice software. Also, Linkage disequilibrium score regression (LD Score regression) analysis was performed to evaluate the genetic correlation between OP and SCZ. Multi-trait analysis of GWAS (MTAG) was performed to detect novel candidate genes for osteoporosis and SCZ.

**Results:**

In the Caucasia discovery samples, significant genetic correlations were observed for ulna & radius BMD vs. SCZ (*P* value = 0.010), ulna & radius area vs. SCZ (*P* value = 0.031). In the Chinese replication samples, we observed significant correlation for ulna & radius area vs. SCZ (*P* value = 0.019). In addition, LD Score regression also identified significant genetic correlations between SCZ and bone phenotypes in Caucasian and Chinese sample respectively. MTAG analysis identified several novel candidate genes, such as CTNNA2 (MTAG *P* value = 2.24 × 10^−6^) for SCZ and FADS2 (MTAG *P* value = 2.66 × 10^−7^) for osteoporosis.

**Conclusions:**

Our study results support the overlapped genetic basis for osteoporosis and SCZ, and provide novel clues for elucidating the biological mechanism of increased osteoporosis risk in SCZ patients.

## Background

Schizophrenia (SCZ) is a psychiatric disorder characterized by delusions, hallucinations and cognitive deficits [1]. The estimated life time risk of SCZ is about 1% in the general population [2]. SCZ usually first occurs in the young adulthood with ages ranging from 16 to 30 years. The clinical manifestations of SCZ mainly include false beliefs, confused thinking, phonism, reduced social engagement and emotional expression. SCZ patients usually have additional mental problems, such as depressive, anxiety and substance-use disorders. It has been demonstrated that SCZ is a highly heritable psychiatric disorder [2, 3]. The estimated heritability of SCZ achieved 31% and 44% for nuclear and extended families [4]. Extensive efforts have been paid to reveal the genetic basis of SCZ as well as it genetic relationships with other diseases or traits, such as amyotrophic lateral sclerosis [5], smoking behaviors [6] and age at first birth in women [7].

Osteoporosis (OP) is a metabolic bone disease mainly characterized by low bone mass and high bone fragility [8, 9]. Osteoporosis is one of the most serious public health problems around the world, resulting in millions of fractures annually, high mortality and excessive therapeutic cost [10, 11]. Bone mineral density (BMD) is the most widely used predictor for osteoporosis with estimated heritability more than 50% [12]. Significant genetic correlations have been observed between osteoporosis and other disorders, such as coronary artery disease [13] and Kashin-Beck disease (KBD) [14].

It has been reported that SCZ patients exhibited increased risk of osteoporosis [15, 16]. This phenomenon was originally attributed to polydipsia and increased urinary calcium excretion in SCZ patients [17]. However, inconsistent results were reported by the following studies [18]. Subsequently, antipsychotics treatment was regarded as an important cause of the correlation between osteoporosis and SCZ [19–21]. But no significant relevance of antipsychotics use with SCZ was also reported in the SCZ patients undergoing antipsychotic treatment for a long time [22]. Additionally, eating disorder, lack of sunlight exposure and insufficient exercise caused by negative symptom were suggested to increase the risk of osteoporosis in SCZ patients [23]. But the biological mechanism underlying the correlation between osteoporosis and SCZ remains elusive now. Given the complicated biological processes implicated in the development of osteoporosis and SCZ, it was suggested that multiple factors should be considered for understanding the relationships between osteoporosis and SCZ [23]. Recently, the genetic correlations of complex diseases are extensively explored, demonstrating the generality of shared genetic basis among various complex disorders [24]. Given the high heritability and clinically observed correlation of osteoporosis and SCZ [4, 12, 15, 16], it is interesting and helpful to investigate the genetic relationship between osteoporosis and SCZ. However, to the best of our knowledge, few efforts have been paid to explore the possible genetic basis shared by osteoporosis and SCZ.

In this study, a trans-ethnic two-stage genetic correlation analysis of osteoporosis and SCZ was first conducted, totally invoking 2286 Caucasian subjects in discovery stage and 4124 Chinese subjects in replication stage. Multi-trait analysis of genome-wide association study (GWAS) approach was then applied to the GWAS summary datasets of osteoporosis and SCZ to detect novel candidate genes for osteoporosis and SCZ. We hope that our study results provide novel clues for understanding the biological mechanism underlying the relationship between osteoporosis and SCZ.

## Materials and methods

### Ethics statement

This study was approved by the Institutional Review Board of Xi’an Jiaotong University and University of Missouri-Kansas City. Signed informed-consent documents were obtained from all study participants before entering the study.

### Caucasians discovery samples for osteoporosis (Caucasian OP)

A total of 2286 unrelated Caucasians subjects living in Kansas City and its surrounding areas were collected. Nurse-administered questionnaires were used to obtain the clinical data of each study subject, including self-reported ethnicities, lifestyle characteristics, health statuses, family and medical histories. We excluded the subjects with chronic diseases, conditions and drug use that might affect bone growth and metabolism. Hologic 4500W dual energy X-ray absorptiometry (Hologic Inc., Bedford, MA, USA) was used to measure the BMD and bone areas at ulna & radius, hip and spine. SNP genotyping was performed using Genome-Wide Human SNP Array 6.0 (Affymetrix, Santa Clara, CA, USA). Arrays were scanned using GeneChip Scanner 3000 7G. For quality control, we excluded the SNPs with Hardy–Weinberg equilibrium (HWE) testing P values < 0.0001 and minor allele frequencies (MAF) < 0.01 and genotyping call rate < 95% in this study. Detailed description of sample characteristics, experimental design and quality control can be found in our previous study [25].

### Chinese replication samples for osteoporosis (Chinese OP)

The Chinese replication GWAS samples of osteoporosis consists of 1627 Han Chinese subjects (including 802 males and 825 females), collecting at Xi’an city and Changsha city of China. Nurse-administered questionnaires were used to obtain the clinical data of each study subject, including self-reported ethnicities, lifestyle characteristics, health statuses, family and medical histories. We excluded the subjects with chronic diseases and conditions that might affect bone growth and metabolism. The BMD and bone area values of ulna & radius, hip and spine were measured using Hologic 4500W dual energy X-ray absorptiometry machine (Hologic Inc., Bedford, MA, USA). Genome-wide SNP genotyping was performed using Genome-Wide Human SNP Array 6.0 (Affymetrix, Santa Clara, CA, USA), and scanned using the GeneChip Scanner 3000 7G. For quality control, the SNPs with HWE testing *P* value < 0.0001, MAF < 0.01 and genotyping call rate < 95% were excluded from this study. Detailed description of sample characteristics, experimental design and quality control can be found in our previous study [26].

### Chinese replication sample for SCZ (Chinese SCZ)

The Chinese SCZ samples were collected at the First Affiliated Hospital of Xi’an Jiaotong University. It consists of 1475 SCZ patients and 1022 unrelated healthy controls. Nurse-administered questionnaires were used to obtain the clinical data of each study subject, including self-reported ethnicities, lifestyle characteristics, health statuses, family and medical histories. SCZ was diagnosed according to according to the Diagnostic and Statistical Manual of Mental Disorders (DSM)-IV criteria by two psychiatrists [27]. We excluded the subjects with other psychiatric disorders, conditions and drug use that might affect mental status. Genome-wide SNP genotyping was performed using Genome-Wide Human SNP Array 6.0 (Affymetrix, Santa Clara, CA, USA), and scanned using the GeneChip Scanner 3000 7G. For quality control, the SNPs with HWE testing *P* value < 0.0001, MAF < 0.01 and genotyping call rate < 95% were excluded from this study.

### GWAS summary dataset 1 of SCZ (GWASSCZ1)

The Caucasian GWAS summary data of SCZ was driven from a large-scale GWAS meta-analysis of SCZ [2]. Briefly, this GWAS dataset comprised of 49 non-overlapping case–control samples (including 34,241 cases and 45,604 controls from 46 cohorts of European and 3 cohorts of East Asian ancestry) and 3 family-based samples (including 1235 parent affected-offspring trios from 17 separate GWAS of European ancestry). Genotyping was originally conducted using commercial platforms, such as Affymetrix 5.0 array, Affymetrix 6.0 array and Illumina 550K array. Genotypes from all studies were processed by the Psychiatric Genomics Consortium (PGC) using unified quality control procedures. Meta-analysis of all individual GWAS was performed using an inverse-weighted fixed effects model. Detailed description of sample characteristics, experimental design, statistical analysis and quality control can be found in the previous study [2].

### Polygenetic risk scoring (PRS) analysis

PRS analysis is a powerful approach and widely applied for detecting shared genetic aetiology among traits [4]. PRSs are an estimate of disease risk conducted by the individual based on the risk alleles and the corresponding effect sizes obtained from the GWAS summary statistics. PRS analysis uses the beta coefficients from the GWAS as weight for each SNP allele in order to calculate an overall risk score for each individual subject in an independent sample.

PRS analysis of Caucasian OP vs. GWASSCZ1 and Chinese OP vs. Chinese SCZ were conducted by the PRSice software, respectively (https://github.com/choishingwan/PRSice) [28]. PRSice performs clumping to remove ambiguous SNPs that are in LD with each other as a default [28]. Furthermore, it can calculate PRS at any number of P-value thresholds in order to provide the most predictive (precise) threshold and best-fit PRS [28]. By utilizing the GWAS results of base traits, and individual genotyping data of target traits, PRSice is capable of calculating the PRS of the base phenotype, and then the calculated PRS were used as the predictors of the target phenotype in a regression model [29].

### Linkage disequilibrium score regression (LD Score regression)

Following the standard approach recommended by the developers, LD Score regression software (https://github.com/bulik/ldsc) was used here to evaluate the genetic correlation between OP and SCZ in Caucasian discovery samples and Chinese replication samples separately. LD Score regression is a powerful approach for genetic correlation analysis of complex diseases or traits [30]. LD Score regression is robust to the confounding biases caused by cryptic relatedness and population stratification. Inflated distribution of test statistics in GWAS can be caused by polygenicity (many small genetic effects) and confounding biases (cryptic relatedness and population stratification) [30]. LD Score regression can quantify these differences and the contribution of each factor by detecting regression relationships between linkage disequilibrium and test statistics [30].

Besides the PRS, LD Score regression provides an alternative way to measure the genetic correlation between BMD and SCZ. In PRS analysis, in order to optimize prediction power, only the significant SNPs were used, while LD Score regression analysis utilized whole genome SNPs data for genetic correlation analysis.

### Multi-trait analysis of GWAS

Utilizing the GWAS summary datasets of Chinese OP and Chinese SCZ, MTAG approach was applied to identify novel candidate genes for osteoporosis and SCZ [31]. MTAG extended the inverse-variance-weighted meta-analysis to jointly analyze the GWAS summary data of multiple traits (https://github.com/omeed-maghzian/mtag). MTGA can integrate the information contained in the GWAS summaries of related traits, and increase the statistical power for detect novel causal loci for each trait analyzed [31]. MTAG will output trait-specific SNP association testing statistics for OP and SCZ, respectively. Real data analysis results demonstrated the good performance of MTAG for identifying novel causal loci for genetically correlated complex diseases [31].

### Functional gene sets enrichment analysis

Identified candidate genes for each pair of SCZ and osteoporosis related traits were subjected to gene set enrichment analysis, implemented by the GENE2FUNC of the FUMA tool. FUMA is a platform [32] that has been widely used to annotate, prioritize, visualize and interpret GWAS findings [33]. For every input gene, GENE2FUNC provides information about tissue specificity, the enrichment of publicly available gene sets, and the expression of different tissue types. Significant gene sets were chosen with an adjusted P value < 0.05.

## Results

Table 1 summarizes the polygenetic risk scoring analysis results both of Caucasian discovery samples and Chinese replication samples. We observed genetic associations between osteoporosis phenotypes (ulna & radius area) and SCZ in both discovery and replication samples, suggesting the common genetic factors shared by osteoporosis and SCZ.

**Table 1:** PRS analysis results of BMD, bone area and SCZ

	Bone phenotype	*P* value^a^
Caucasian OP vs. GWASSCZ1	Hip total area	0.311
	Hip total BMD	0.376
	Hip neck area	0.253
	Hip neck BMD	0.276
	Spine area	0.571
	Spine BMD	0.056
	Ulna & radius area	***0.031***
	Ulna & radius BMD	***0.010***
Chinese OP vs. Chinese SCZ	Hip total area	0.349
	Hip total BMD	0.061
	Hip neck area	0.094
	Hip neck BMD	0.061
	Spine area	0.369
	Spine BMD	0.274
	Ulna & Radius Area	***0.019***
	Ulna & radius BMD	0.303

The bolditalic values showed the *P* value ≤ 0.05, which was significant in our study

^a^The *P* value were calculated by PRSice software

In the Caucasian discovery samples (Caucasian OP vs. GWASSCZ1), significant genetic correlations were observed for ulna & radius BMD vs. SCZ (*P* value = 0.010) and ulna & radius area vs. SCZ (*P* value = 0.031). In the replication study of Chinese OP vs. Chinese SCZ, significant correlation was observed for ulna & radius area vs. SCZ (*P* value = 0.019), as well as suggestive correlation signals for Hip total BMD vs. SCZ (*P* value = 0.061) and Hip neck BMD vs. SCZ (P value = 0.061).

For LD Score regression analysis, as expected, we found significant genetic correlation between hip total BMD and SCZ (*P* value = 0.0272) in Caucasians discovery samples. In Chinese replication samples, two significant genetic correlation signals were also observed, including ulna & radius BMD vs. SCZ (*P* value = 0.0042), whole body BMD vs. SCZ (*P* value = 0.0027).

To detect novel candidate genes for osteoporosis and SCZ, we conducted MTAG analysis utilizing the Chinese GWAS summarizes of BMD, bone and SCZ. For each pair of SCZ and osteoporosis related traits, MTGA output SNP association testing statistics for each analyzed trait, respectively. MTAG analysis results were summarized in Table 2. For osteoporosis-related traits, the most strong association signal was observed at the rs174577 of FADS2 gene (MTAG *P* value = 2.66 × 10^−7^). For SCZ, the most strong association signal was observed at the rs17018359 of CTNNA2 gene (MTAG *P* value = 2.24 × 10^−6^).

**Table 2:** Multi-trait analysis results of GWAS for BMD, bone area and SCZ (*P* value < 1.00 × 10^−5^)

Phenotype	SNP	MAF	Beta^a^	*P* value^a^	Gene
Hip total area vs. SCZ					
SCZ	rs17018359	0.23	− 0.101	3.44 × 10^−6^	CTNNA2
SCZ	rs1561296	0.27	− 0.093	7.01 × 10^−6^	LOC101927741/SPATS2L
Hip total BMD vs. SCZ					
SCZ	rs17018359	0.23	− 0.102	3.04 × 10^−6^	CTNNA2
SCZ	rs1561296	0.27	− 0.093	7.53 × 10^−6^	LOC101927741/SPATS2L
Hip neck Area vs. SCZ					
SCZ	rs17018359	0.23	− 0.103	2.81 × 10^−6^	CTNNA2
SCZ	rs1561296	0.27	− 0.093	7.38 × 10^−6^	LOC101927741/SPATS2L
Hip neck BMD vs. SCZ					
Hip neck BMD	rs11259498	0.09	− 0.306	1.06 × 10^−6^	LOC105376431 NMT2
SCZ	rs17018359	0.23	− 0.102	3.09 × 10^−6^	CTNNA2
SCZ	rs1561296	0.27	− 0.093	7.24 × 10^−6^	LOC101927741/SPATS2L
Spine area vs. SCZ					
Spine area	rs174577	0.41	− 0.175	2.66 × 10^−7^	
Spine area	rs174570	0.41	− 0.172	4.85 × 10^−7^	
Spine area	rs174547	0.42	− 0.170	5.31 × 10^−7^	
Spine area	rs8019455	0.22	− 0.189	2.87 × 10^−6^	
SCZ	rs17018359	0.23	− 0.099	4.54 × 10^−6^	
Spine BMD vs. SCZ					
Spine BMD	rs4721564	0.38	− 0.161	8.37 × 10^−6^	
SCZ	rs17018359	0.23	− 0.102	2.24 × 10^−6^	CTNNA2
SCZ	rs16945898	0.1	− 0.140	4.19 × 10^−6^	
SCZ	rs1561296	0.27	− 0.092	6.70 × 10^−6^	LOC101927741/SPATS2L
SCZ	rs1791244	0.43	− 0.083	7.17 × 10^−6^	MIR924HG
Ulna & radius area vs. SCZ					
Ulna & radius area	rs742715	0.43	− 0.180	9.30 × 10^−7^	LOC105377865
SCZ	rs17018359	0.23	− 0.102	3.05 × 10^−6^	CTNNA2
SCZ	rs1561296	0.27	− 0.093	7.55 × 10^−6^	LOC101927741/SPATS2L

^a^The beta and P value were calculated by MTAG

In addition, we performed FUMA analysis for seven pairs of SCZ and osteoporosis related traits, including Hip total area vs. SCZ, Hip total BMD vs. SCZ, Hip neck Area vs. SCZ, Hip neck BMD vs. SCZ, Spine area vs. SCZ, Spine BMD vs. SCZ, ulna & radius area vs. SCZ, respectively. FUMA analysis detected multiple candidate gene sets or pathways for the identified seven associations (Additional file 1: Table S1, Additional file 2: Table S2, Additional file 3: Table S3, Additional file 4: Table S4, Additional file 5: Table S5, Additional file 6: Table S6, Additional file 7: Table S7). Among them, the results of Kyoto Encyclopedia of Genes and Genomes (KEGG) pathway were summarized at Table 3.

**Table 3:** FUMA gene set enrichment analysis results of KEGG pathways for identified associations (Adjusted P value < 0.05)

Name	P value	Adjusted P value
Hip area vs. SCZ		
KEGG_AXON_GUIDANCE	1.686 × 10^−4^	3.136 × 10^−2^
Hip total BMD vs. SCZ		
KEGG_AXON_GUIDANCE	8.719E × 10^−5^	1.622 × 10^−2^
Hip neck Area vs. SCZ		
KEGG_GAP_JUNCTION	2.239 × 10^−4^	2.157 × 10^−2^
KEGG_CALCIUM_SIGNALING_PATHWAY	3.006 × 10^−4^	2.157 × 10^−2^
KEGG_GNRH_SIGNALING_PATHWAY	3.480 × 10^−4^	2.157 × 10^−2^
Hip neck BMD vs. SCZ		
KEGG_ASCORBATE_AND_ALDARATE_METABOLISM	9.782 × 10^−7^	1.462 × 10^−4^
KEGG_PENTOSE_AND_GLUCURONATE_INTERCONVERSIONS	1.572 × 10^−6^	1.462 × 10^−4^
KEGG_PORPHYRIN_AND_CHLOROPHYLL_METABOLISM	7.543 × 10^−6^	4.677 × 10^−4^
KEGG_DRUG_METABOLISM_OTHER_ENZYMES	1.818 × 10^−5^	7.310 × 10^−4^
KEGG_STARCH_AND_SUCROSE_METABOLISM	1.965 × 10^−5^	7.310 × 10^−4^
KEGG_STEROID_HORMONE_BIOSYNTHESIS	2.458 × 10^−5^	7.621 × 10^−4^
KEGG_RETINOL_METABOLISM	4.485 × 10^−5^	1.192 × 10^−3^
KEGG_METABOLISM_OF_XENOBIOTICS_BY_CYTOCHROME_P450	6.381 × 10^−5^	1.473 × 10^−3^
KEGG_DRUG_METABOLISM_CYTOCHROME_P450	7.127 × 10^−5^	1.473 × 10^−3^
Spine area vs. SCZ		
KEGG_NEUROACTIVE_LIGAND_RECEPTOR_INTERACTION	3.722 × 10^−6^	6.922 × 10^−4^
KEGG_NON_SMALL_CELL_LUNG_CANCER	1.615 × 10^−5^	1.501 × 10^−3^
KEGG_ARRHYTHMOGENIC_RIGHT_VENTRICULAR_CARDIOMYOPATHY_ARVC	9.799 × 10^−5^	6.076 × 10^−3^
KEGG_CALCIUM_SIGNALING_PATHWAY	4.238 × 10^−4^	1.971 × 10^−2^
KEGG_VALINE_LEUCINE_AND_ISOLEUCINE_DEGRADATION	9.590 × 10^−4^	3.567 × 10^−2^
KEGG_GLYCEROLIPID_METABOLISM	1.440 × 10^−3^	4.107 × 10^−2^
KEGG_ECM_RECEPTOR_INTERACTION	1.545 × 10^−3^	4.107 × 10^−2^
KEGG_DILATED_CARDIOMYOPATHY	2.096 × 10^−3^	4.874 × 10^−2^
Ulna & radius area vs. SCZ		
KEGG_NEUROACTIVE_LIGAND_RECEPTOR_INTERACTION	1.492 × 10^−4^	2.775 × 10^−2^

## Discussions

To the best of our knowledge, few efforts have been paid to investigate the genetic relationships between osteoporosis and SCZ. In our study, a trans-ethnic two-stage genetic correlation analysis of osteoporosis OP and SCZ was performed. In the first stage, we conducted a PRS analysis to test the association between SCZ and OP in Caucasian discovery samples and replicated in Chinese replication samples. In short, we calculated SCZ-PRS to predict OP status in Caucasian discovery samples while used OP-PRS to predict SCZ status in Chinese replication samples. PRS analysis is a powerful approach, and widely used in current genetic studies of human complex diseases or traits [34, 35]. In addition, we also perform the LD score regression analysis, a genome-wide genetic correlation analysis, between BMD and SCZ in Chinese and Caucasian subject, respectively. In the second stage, we performed MTAG [31] analysis to detect novel candidate genes for OP and SCZ, respectively. And then these identified candidate genes for each pair of SCZ and osteoporosis related traits were subjected to gene set enrichment analysis via FUMA. We hope that our study results provide novel clues for understanding observed clinical correlation between osteoporosis and SCZ.

It has been a long time that researchers observed increased risks of osteoporosis in SCZ patients [15, 16]. Different hypothesis have been proposed, such as antipsychotics treatment, lack of sunlight exposure and insufficient exercise [23]. However, to the best of our knowledge, limited studies were conducted to figure out the potential genetic relationship between osteoporosis and SCZ. In this study, we observed genetic correlations between multiple osteoporosis-related traits and SCZ, suggesting the common genetic mechanism shared by osteoporosis and SCZ.

SNPs that were common in two or more populations often differed significantly in frequency between populations [36]. Even if alleles have similar frequencies among different populations, the effects of alleles on risk might be specific to certain populations. For causal variants, there are many reasons for the heterogeneity of genetic effects across populations, including clinical heterogeneity, differences in pathophysiology, gene–environment interaction and gene–gene interactions [37]. Recently, Lam et al. and his colleagues compared the genetic architectures of SCZ in two major world populations-East Asian and European samples, and the results indicating the common variant genetic architecture of SCZ outside of the major histocompatibility complex (MHC) region is highly consistent across East Asian and European populations [37]. Nevertheless, they also found the polygenic risk calculated for East Asians are different from the Europeans, which can be explained by ancestry-related differences in allele frequencies, LD and other factors [37]. In addition, existing studies showed there are some differences for osteoporosis between East and West. For example, the secular changes in hip fracture rates differed between East and West, with rates currently declining in North America, Oceana, Northern Europe, Hong Kong, Taiwan, and in most of Central Europe, but with increasing rates of hip fractures in much of Asia, Southern Europe, and South America, indicating close relationships between rising rates of urbanization and hip fractures across disparate geographic locations and cultures [38]. Another study identified significantly differentially expressed genes in circulating monocytes between the high and low BMD groups in Chinese Han females and validated the significant expression in Caucasian women, and they found STAT1 gene was upregulated in the low and the high BMD groups in both Chinese and Caucasians [39]. Furthermore, they also found COL1A1 gene may have significantly association with bone geometry in both Caucasians and Chinese, but AHSG gene may be linked to bone geometry in Caucasians, not in Chinese [40]. These evidences showed there might be a similar pattern between Chinese population and West population. In this study, we observed the genetic correlation of OP-related phenotypes and SCZ in Caucasians population and validated it in the Chinese population.

The potential molecular mechanism of the genetic correlation between osteoporosis and SCZ was largely unknown now. Recent studies may provide some helpful clues. For instance, it is well known that vitamin D and vitamin D metabolism related genes play import roles in the development of bone and osteoporosis [41]. Valipour et al. observed strong association between serum vitamin D level and schizophrenia [42]. Amato et al. detected significant overlap between vitamin D metabolism related genes and SCZ associated genes [43]. Additionally, Beydoun et al. found that vitamin D status was negatively correlated with cognitive decline in US urban adults [44]. Coşkun et al. observed significant association between vitamin D receptor gene polymorphism and the risk of autism spectrum disorder [45]. Mokry et al. evaluated the impact of vitamin D metabolism associated genes on the risks of Alzheimer disease [46]. Their genetic analysis results support 25-hydroxyvitamin D as a causal risk factor for Alzheimer disease [46].

The genetic correlation between osteoporosis and SCZ may also attribute to the exposure of common environment risk factors, such cigarette smoking and alcohol drinking, which are under genetic control. The significant impact of cigarette smoking and alcohol drinking on the development of osteoporosis has been well documented [47]. Furthermore, GWAS found that the risk of schizophrenia was associated with a cluster of genes (CHRNA5, CHRNA3 and CHRNB5) on chromosome 15 [2], which was also suggested to be associated with both early age at onset of cigarette smoking and heavy cigarette smoking [48, 49]. Alcohol drinking disorder is the most common co-occurring disorder in the people with SCZ [50]. GWAS also detected alcohol drinking associated genetic loci, the most robust ones of which were in GABAergic system GABRA2 and alcohol-metabolizing enzymes ALDH2, ADH1B, and ADH1C [51, 52]. Zai et al. suggested that neural plasticity and transmission related genes BDNF and DRD3 gene polymorphisms were significantly associated with alcohol drinking in SCZ patients [53].

MTAG analysis also identified several candidate genes for osteoporosis and SCZ. For instance, we found that CTNNA2 was associated with SCZ in this study. CTNNA2 encodes catenin alpha 2 protein, which was suggested as a candidate genes for SCZ [54]. CTNNA2 is critical for maintaining the stability of dendritic spines in rodent hippocampal neurons [55]. CTNNA2 knockout mice exhibited hippocampal pyramidal cell disorganization and deficits in prepulse inhibition of the startle response, which were also observed in SCZ patients [56]. It is more interesting that cigarette smoking was capable of regulating the expression of CTNNA2 in SCZ patients [57].

In this study, we aimed to explain the relationship between SCZ and OP from the perspective of genetics, which was usually not affected by the environmental confounding factors. Using the powerful PRS and MTAG approaches, we explored the genetic association of SCZ and OP in both Caucasian discovery samples and Chinese replication samples. Furthermore, the large sample sizes and discovery-replication study design ensured the accuracy of our study results. As far as we know, this is the first systemic study exploring the potential association between SCZ and OP from the genetic term, and this is the main difference between our study and previous study.

Several issues of this study should be noted. First, the Chinese replication sample sizes for osteoporosis (1627 subjects) and SCZ (2497 subjects) was relatively small in the MTAG analysis. The candidate genes identified by MTAG for osteoporosis and SCZ should be interpreted with caution. Second, the observed genetic correlations between osteoporosis and SCZ appeared to be skeletal site specific, mainly limited to ulna & radius and hip. Skeletal site specific effects of genetic factors on skeletal growth & development and osteoporosis had been demonstrated in previous genetic studies [58, 59]. Also, as an important limitation of our study, the target molecules identified in our study should be validated in mechanism-based studies. In the further analysis, cells as well as animal experiments and annotation analysis are warranted to confirm our finding and clarify the biological mechanism of the identified molecules contributing to the development of SCZ and OP in our study.

In conclusion, we conducted a trans-ethnic two-stage genetic correlation analysis of osteoporosis and SCZ. We observed genetic correlations between multiple osteoporosis related traits and SCZ, suggesting the overlapped genetic mechanism shared by osteoporosis and SCZ. We hope that our study results may help to elucidate the mechanism of increased osteoporosis risk in SCZ patients.

## Supplementary information

**Additional file 1: Table S1.** FUMA gene set enrichment analysis results for the association between hip total area and SCZ.

**Additional file 2: Table S2.** FUMA gene set enrichment analysis results for the association between hip neck area and SCZ.

**Additional file 3: Table S3.** FUMA gene set enrichment analysis results for the association between hip neck BMD and SCZ.

**Additional file 4: Table S4.** FUMA gene set enrichment analysis results for the association between hip total BMD and SCZ.

**Additional file 5: Table S5.** FUMA gene set enrichment analysis results for the association between spine area and SCZ.

**Additional file 6: Table S6.** FUMA gene set enrichment analysis results for the association between spineBMD and SCZ.

**Additional file 7: Table S7.** FUMA gene set enrichment analysis results for the association between Ulna & Radius area and SCZ.

## Data Availability

The datasets used and/or analysed during the current study are available from the corresponding author on reasonable request.

## Abbreviations

SCZ: Schizophrenia
OP: Osteoporosis
GWAS: Genome-wide association study
MTAG: Multi-trait analysis of GWAS
BMD: Bone mineral density
MAF: Minor allele frequencies
PGC: Psychiatric Genomics Consortium
PRSice: Polygenic risk score software
